# Supplementary material for: Unveiling the cell-type-specific landscape of cellular senescence through single-cell transcriptomics using SenePy
Source: Nat Commun. 2025 Feb 22;16:1884. doi: 10.1038/s41467-025-57047-7 (PMC11846890; doi:10.1038/s41467-025-57047-7)
Supplement: Supplementary file 1 — Supplementary Information [file 41467_2025_57047_MOESM1_ESM.pdf]

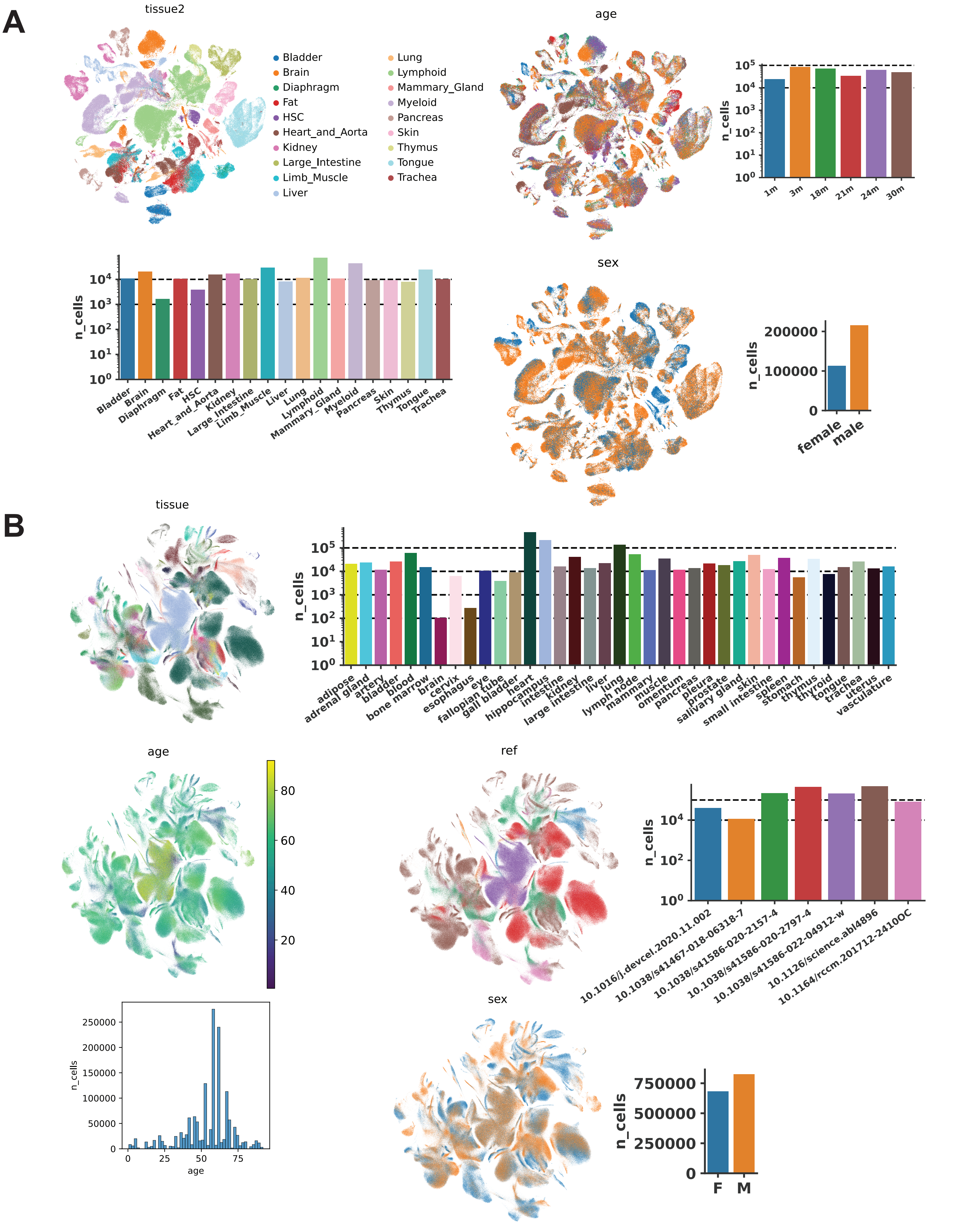

Supplemental figure 1. Dataset description (**A, B**) Descriptive UMAPs and barplots of all mouse and human cells used, which are colored by tissue of origin, age, and sex.

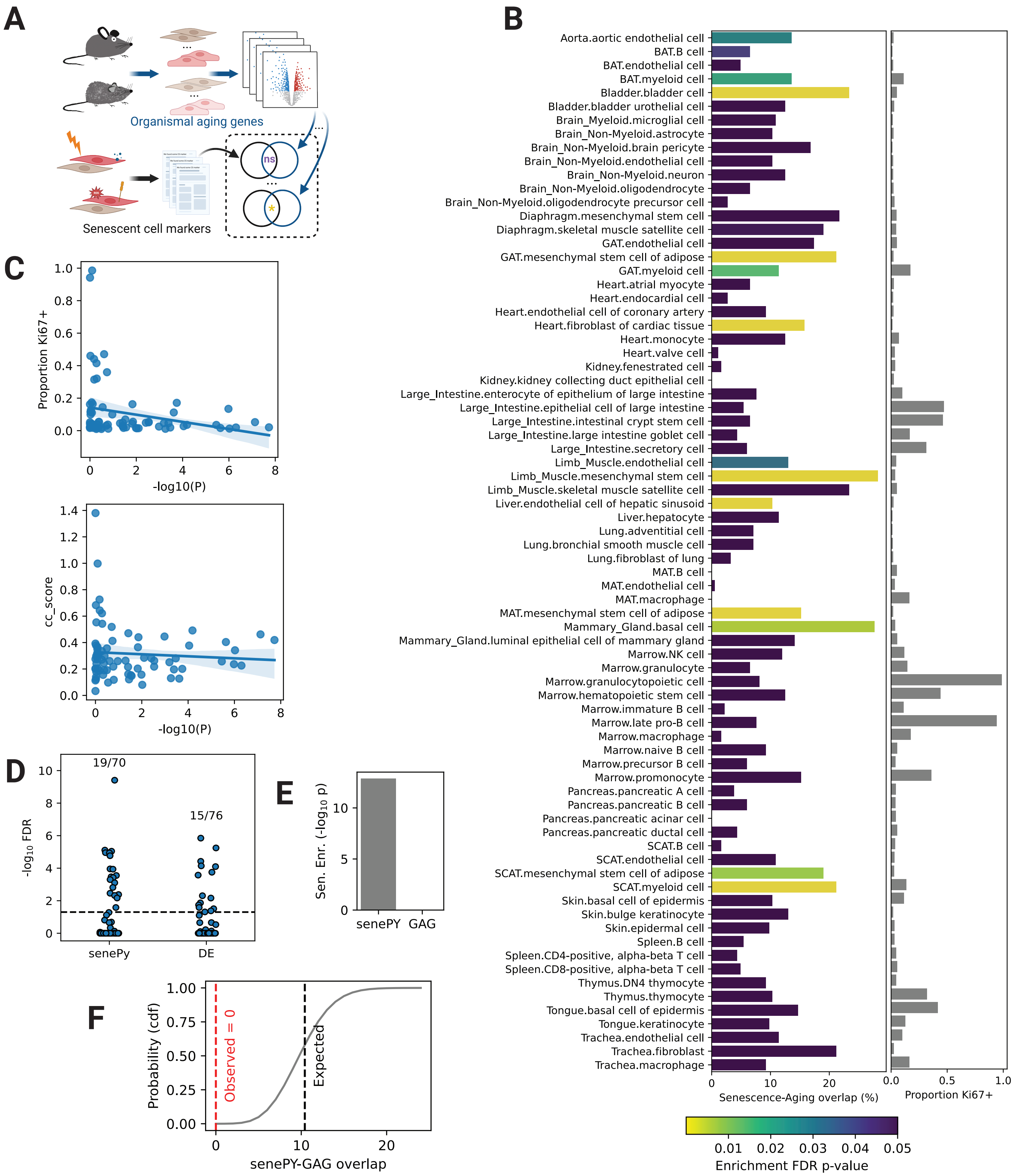

Supplemental figure 2. Traditional methods poorly characterize in vivo senescence (**A**) Overview of the senescence marker gene set enrichment analysis. Cell-specific organismal aging signatures determined in a previous study were compared to a curated set of senescence markers taken from multiple studies. Created in BioRender. Rehman, J. (2025) https://BioRender.com/l74y283 (**B**) The statistical enrichment of every organismal aging signature for senescence markers. Color represents the significance of enrichment (Hypergeometric, BH-corrected) and the size of the bar represents the percentage of senescence markers present in the aging signature. The aligned plot on the right shows the proportion of cells in each population that were expressing the proliferation marker Ki-67. (**C**) There is a negative correlation (Pearson's R) between senescence gene set enrichment and the proliferation state of a population based on Ki-67 (top) and cell-cycle scores (bottom). (**D**) The novel senePy signatures enriched for known markers of CS versus organismal aging signatures derived from differential expression. (**E**) The enrichment of the universal senePy mouse signature and the differential expression-based global aging gene set (GAG) for known markers of CS. (**F**) The observed versus expected overlap of the senePy universal signature and the GAG set.

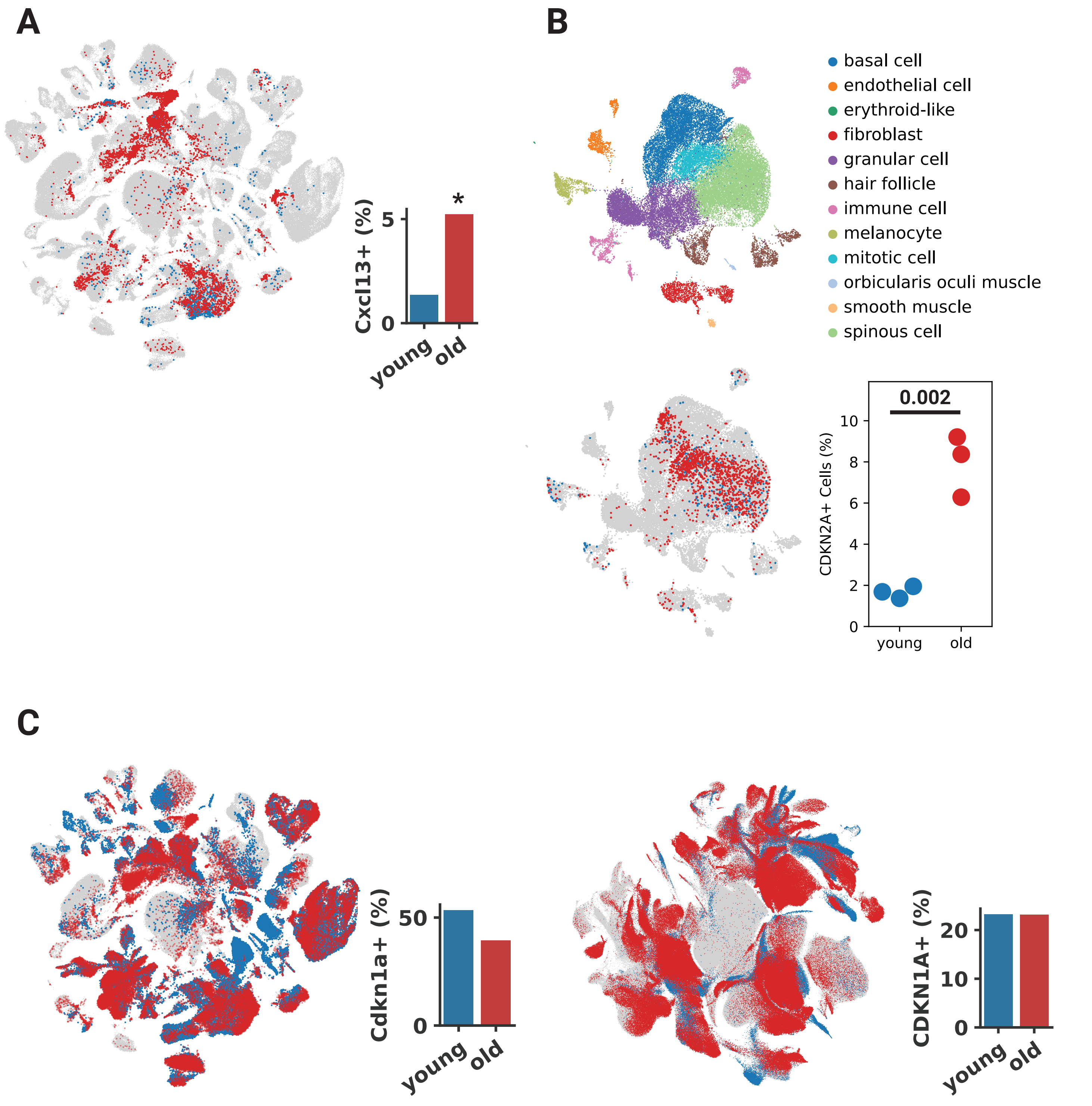

Supplemental Figure 3. Aging dynamics of common cellular senescence markers (**A**) UMAP representing Cxcl13+ cells in all mouse cells. Statistical significance is based on random permutation (* = FDR p-value < 0.05). (**B**) UMAP (top) of human skin cells colored by cell type. UMAP (bottom) of human skill cells positive for CDKN2A from young (age 18-34) and old (age 34-58) donors. Dot plot depicts the percentage of CDKN2A+ cells from old and young human skin cells. (**C**) UMAP representing CDKN1A+ (gene that encodes p21cip1) cells in all mouse and human cells.

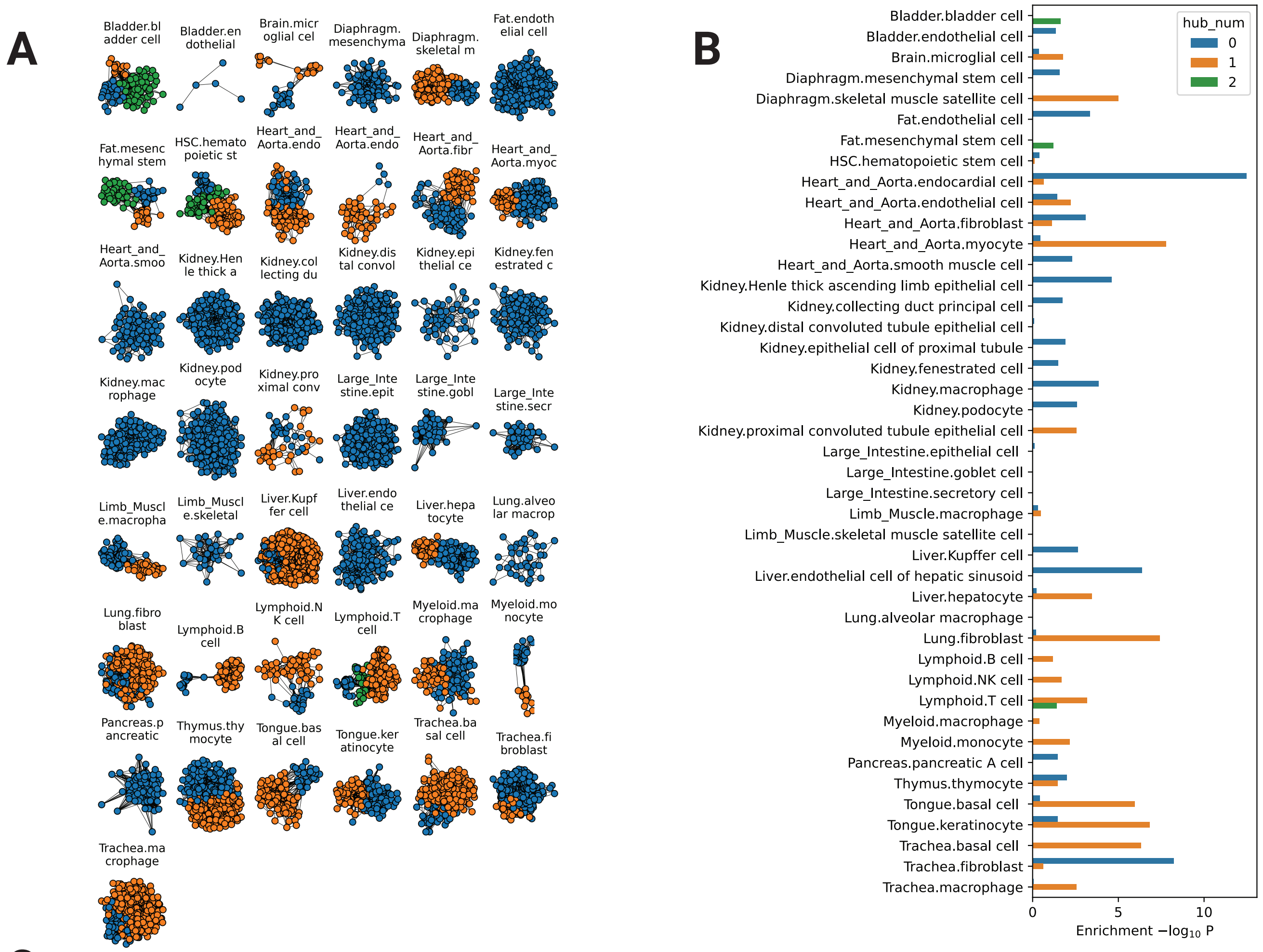

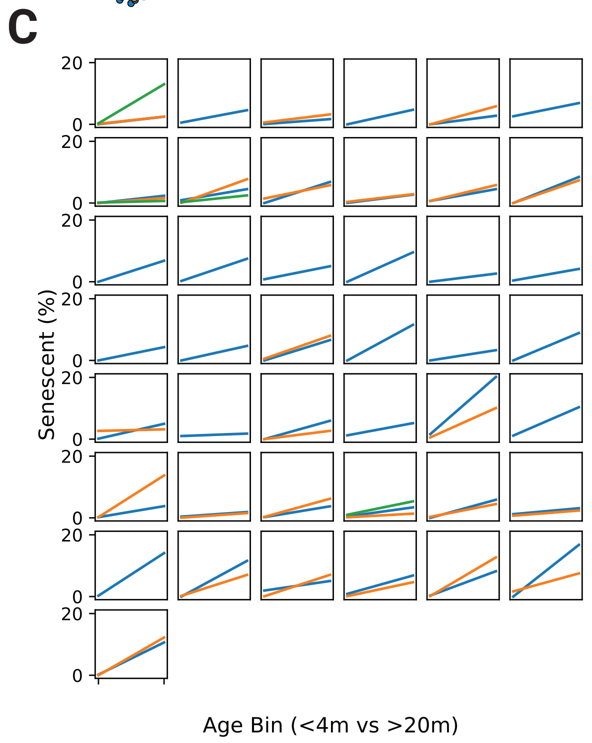

Supplemental Figure 4. Description and kinetics of senePy gene signatures (**A**) SenePy gene binary coexpression networks for every mouse signature. Colors note different hubs within the overall cell networks. Each node represents a gene and each line is a significant coexpression between them. (**B**) Enrichment of known cellular senescence markers in each gene hub. (**C**) The proportion of cells in young and old organisms that express these signatures in their respective cell type. The position of the plots matches figure A and the colors denote the specific gene correlation hub. Cells were binned between young (< 4 month) and old (> 20 month) mice.

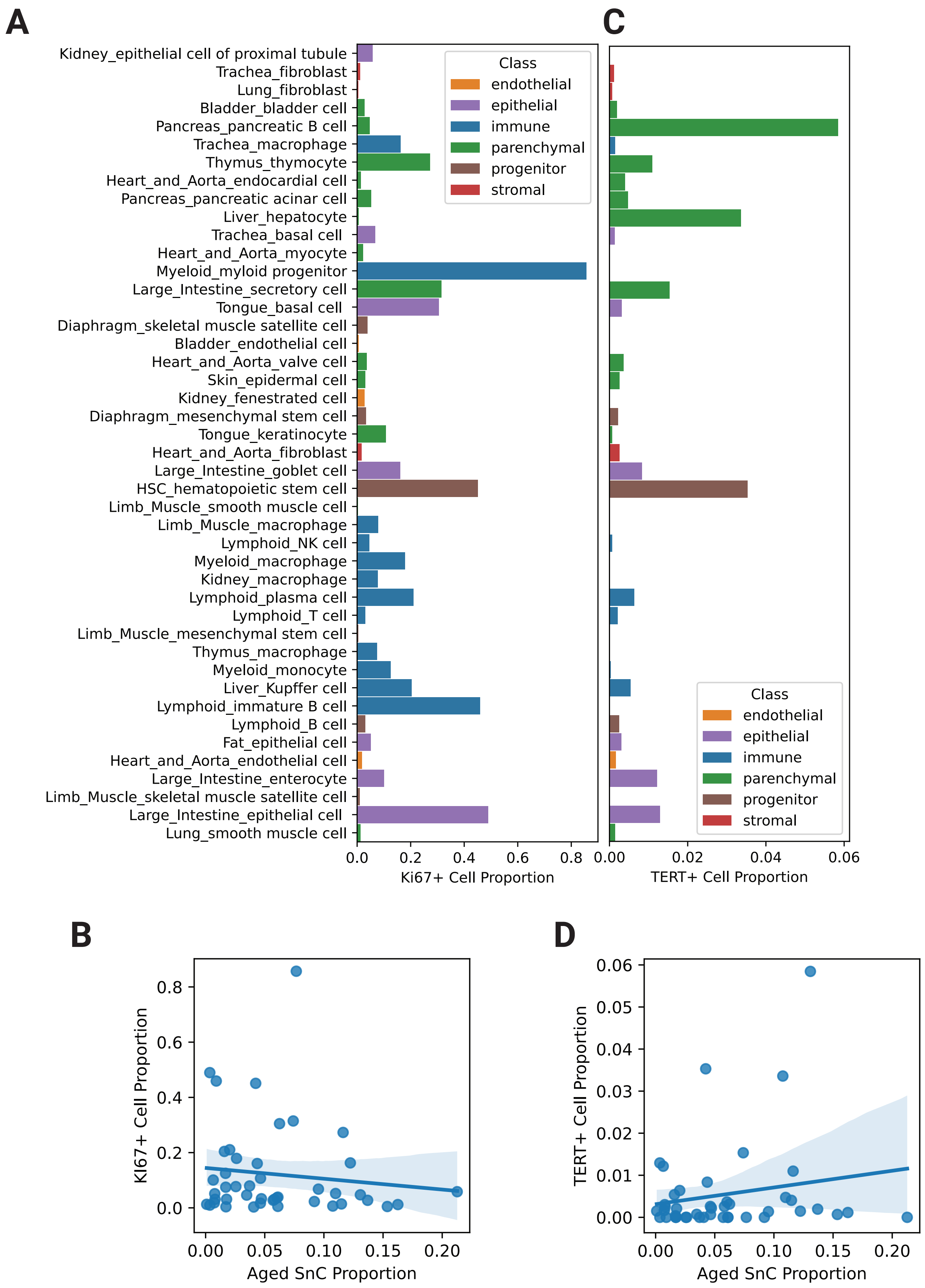

Supplemental Figure 5. Relationship between cell proliferation and senescent cell accumulation (**A**) The proportion of mouse cells expressing Tert and (**B**) Ki67. (**C,D**) The relationship between cell types expressing Tert and Ki67 with their gain in SenePy identified cells.

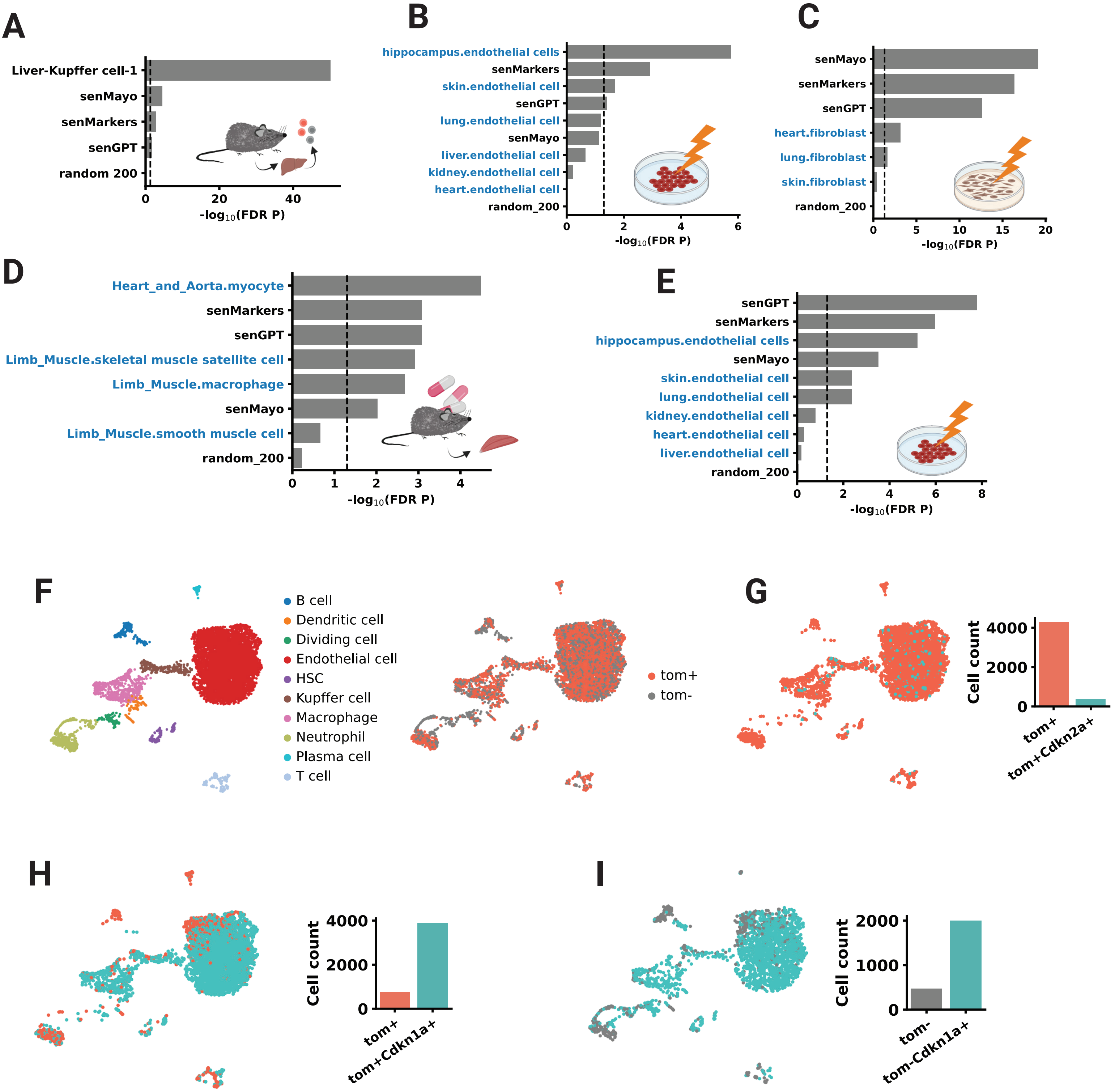

Supplemental Figure 6. Benchmarking senePy in cellular senescence datasets. (**A**) Enrichment analysis of differentially abundant genes in the td-Tomato+ liver cells. Gene sets were derived in this study and we also include the SenMayo signature, CS markers from the literature (senMarkers), and a CS gene set derived from Chat-GPT4 (senGPT). (**B**) Enrichment analysis of gene mRNAs more abundant in irradiated endothelial cells and (**C**) fibroblasts. (**D**) Enrichment analysis of gene mRNAs less abundant in mouse muscle tissue after treatment with a senolytic. Blue lables indicate SenePy signatures. (**E**) Enrichment analysis of gene mRNAs more abundant after irradiation induced senescence of cultured endothelial cells. (**F**) UMAPs of single-cells from the liver which were enriched for td-Tomato+ cells. (**G**) UMAP and representative bar graph of td-Tomato+ cells with Cdkn2a+ cells labeled. (**H**) UMAPs and representative bar graphs of td-Tomato+ and (**I**) td-Tomato- cells with Cdkn1a+ cells labeled. The annotation icons in A-E were created in BioRender. Sanborn, M. (2025) https://BioRender.com/l04t362.

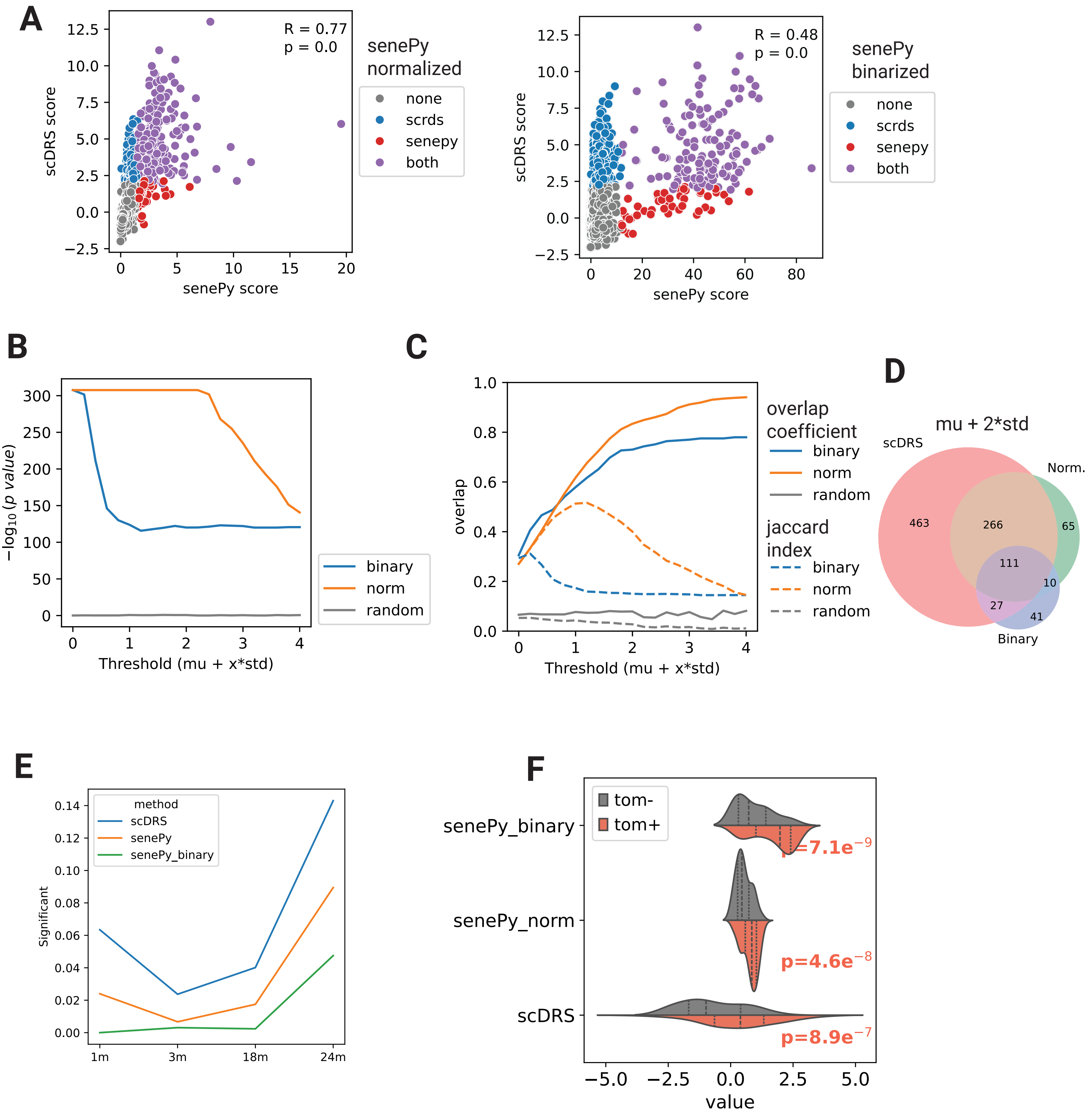

Supplemental Figure 7. Comparing senePy scoring methods. Mouse tongue keratinocytes were scored with a senePy tongue keratinocyte hub using either the senePy scoring method or scDRS (**A-E**). (**A**) Correlation between scDRS scores and senePy default binarized scoring (right) or senePY scoring based on normalized counts (left). (**B**) Overlap statistical significance (Hypergeometric) between the cells identified by scDRS and senePY along varying thresholds. (**C**) Overlap coefficient and Jaccard similarity of the overlap between scDRS and senePY along varying thresholds. (**D**) The overlap between all three methods with a senePy threshold of 2 standard deviations above the mean. (**E**) The proportion of cells identified to be expressing the gene set at varying mouse ages. (**F**) Score distributions in the Td-tomato p16 reporter mouse kidney cells (Mann-Whitney).
