# Supplementary material for: Unveiling the cell-type-specific landscape of cellular senescence through single-cell transcriptomics using SenePy
Source: Nat Commun. 2025 Feb 22;16:1884. doi: 10.1038/s41467-025-57047-7 (PMC11846890; doi:10.1038/s41467-025-57047-7)
Supplement: Supplementary file 2 — Description of Supplementary Data 1-9 [file 41467_2025_57047_MOESM2_ESM.docx]

File Name: Supplementary Data (Supplementary Data 1)

Description: Dynamics of cellular senescence markers in various mouse cell types with organismal age. Each row represents a specific tissue, cell, and gene. Ending and gain represent percentage points. Gene count is the number of dynamic senescence gene in the respective cell type. Cell count is the number of cells types the respective gene is found to be dynamic in. The p value is calculated by random permutation of the data.

File Name: Supplementary Data (Supplementary Data 2)

Description: Dynamics of cellular senescence markers in various human cell types with organismal age. Each row represents a specific tissue, cell, and gene. Ending and gain represent percentage points. Gene count is the number of dynamic senescence gene in the respective cell type. Cell count is the number of cells types the respective gene is found to be dynamic in. The p value is calculated by random permutation of the data.

File Name: Supplementary Data (Supplementary Data 3)

Description: Genes (rows) of every individual mouse cell signature (columns). 1 denotes that the gene is in the signature and 0 means it is not. Signatures represent cell-specific *senePy* signatures.

File Name: Supplementary Data (Supplementary Data 4)

Description: Genes (rows) of every individual human cell signature (columns). 1 denotes that the gene is in the signature and 0 means it is not. Signatures represent cell-specific *senePy* signatures.

File Name: Supplementary Data (Supplementary Data 5)

Description: Genes (rows) of every individual mouse cell-hub signature (columns). 1 denotes that the gene is in the signature and 0 means it is not. Signatures represent cell-specific *senePy* signatures separated into individual gene expression hubs.

File Name: Supplementary Data (Supplementary Data 6)

Description: Genes (rows) of every individual human cell-hub signature (columns). 1 denotes that the gene is in the signature and 0 means it is not. Signatures represent cell-specific *senePy* signatures separated into individual gene expression hubs.

File Name: Supplementary Data (Supplementary Data 7)

Description: Univeral senePy mouse signature. P-value is the chance the gene would occur in the respective number of signatures (“Count”) by chance. Count is the number of individual signatures the respective gene was found in. See methods section "Merging multiple signatures and identifying a universal senescence signature" for information of p-value calculation. Q value is a BH correction.

File Name: Supplementary Data (Supplementary Data 8)

Description: Genes that were overrepresented in mouse endothelial cells, fibroblasts, and macrophage senePy signatures. Count is the number of individual signatures the respective gene was found in. See methods section "Merging multiple signatures and identifying a universal senescence signature" for information of p-value calculation. Q value is a BH correction.

File Name: Supplementary Data (Supplementary Data 9)

Description: Univeral senePy human signature. P-value is the chance the gene would occur in the respective number of signatures (“Count”) by chance. Count is the number of individual signatures the respective gene was found in. See methods section "Merging multiple signatures and identifying a universal senescence signature" for information of p-value calculation. Q value is a BH correction.
